# Levels of human replication factor C4, a clamp loader, correlate with tumor progression and predict the prognosis for colorectal cancer

**DOI:** 10.1186/s12967-014-0320-0

**Published:** 2014-11-19

**Authors:** Jun Xiang, Lekun Fang, Yanxin Luo, Zuli Yang, Yi Liao, Ji Cui, Meijin Huang, Zihuan Yang, Yan Huang, Xinjuan Fan, Huashe Wang, Lei Wang, Junsheng Peng, Jianping Wang

**Affiliations:** Department of Gastrointestinal Surgery, The Sixth Affiliated Hospital, Sun Yat-sen University, 26 Yuancun Er Heng Rd., Guangzhou, 510655 Guangdong China; Guangdong Institute of Gastroenterology, The Sixth Affiliated Hospital, Sun Yat-sen University, 26 Yuancun Er Heng Rd., Guangzhou, 510655 Guangdong China; Department of Colorectal Surgery, The Sixth Affiliated Hospital, Sun Yat-sen University, 26 Yuancun Er Heng Rd., Guangzhou, 510655 Guangdong China; Guangdong Key Laboratory of Colorectal and Pelvic Floor Diseases, The Sixth Affiliated Hospital, Sun Yat-sen University, 26 Yuancun Er Heng Rd., Guangzhou, 510655 Guangdong China; Department of Gastrointestinal Surgery, The First Affiliated Hospital, Sun Yat-sen University, 58 Zhongshang Er Rd., Guangzhou, 510080 Guangdong China; Department of Pathology, The Sixth Affiliated Hospital, Sun Yat-sen University, 26 Yuancun Er Heng Rd., Guangzhou, 510655 Guangdong China

**Keywords:** Colorectal cancer, Replication factor C4, Prognosis, Cell cycle

## Abstract

**Background:**

Human replication factor C4 (RFC4) is involved in DNA replication as a clamp loader and is aberrantly regulated across a range of cancers. The current study aimed to investigate the function of RFC4 in colorectal cancer (CRC).

**Methods:**

The mRNA levels of *RFC4* were assessed in 30 paired primary CRC tissues and matched normal colonic tissues by quantitative PCR. The protein expression levels of *RFC4* were evaluated by western blotting (n = 16) and immunohistochemistry (IHC; n = 49), respectively. Clinicopathological features and survival data were correlated with the expression of RFC4 by IHC analysis in a tissue microarray comprising 331 surgically resected CRC. The impact of RFC4 on cell proliferation and the cell cycle was assessed using CRC cell lines.

**Results:**

*RFC4* expression was significantly increased in CRC specimens as compared to adjacent normal colonic tissues (*P* <0.05). High levels of RFC4, determined on a tissue microarray, were significantly associated with differentiation, an advanced stage by the Tumor-Node-Metastasis (TNM) staging system, and a poor prognosis, as compared to low levels of expression (*P* <0.05). However, in multivariate analysis, RFC4 was not an independent predictor of poor survival for CRC. In *vitro* studies, the loss of RFC4 suppressed CRC cell proliferation and induced S-phase cell cycle arrest.

**Conclusion:**

*RFC4* is frequently overexpressed in CRC, and is associated with tumor progression and worse survival outcome. This might be attributed to the regulation of CRC cell proliferation and cell cycle arrest by RFC4.

**Electronic supplementary material:**

The online version of this article (doi:10.1186/s12967-014-0320-0) contains supplementary material, which is available to authorized users.

## Introduction

Colorectal cancer (CRC) is one of the leading causes of cancer-related deaths worldwide. CRC arises through the accumulation of genetic mutations and epigenetic alterations that result in the transformation of normal colon epithelial cells into adenomas, which may be the precursor of CRC, and ultimate into cancers. Alongside the “normal → adenoma → cancer” pathological progression, numerous molecular alterations have been identified [1,2]. Understanding the underlying molecular mechanisms of CRC might help guide clinical therapies. For example, current clinical guidelines recommend checking the KRAS mutation status before treating CRC patients with EGFR inhibitors.

Human replication factor C (RFC) is a multimeric protein consisting of five distinct subunits that are highly conserved through evolution [3]. The RFC family functions as clamp loaders that load PCNA onto DNA in an ATP-dependent process during DNA synthesis [4-7]. In addition, RFCs play an important role in DNA repair activities following DNA damage [8,9]. Among the RFCs, the *RFC4* gene, that encodes the fourth largest subunit of the RFC complex, has been reported by cDNA microarray or meta-analysis to be deregulated in diverse malignancies, including prostate cancer, cervical cancer, and head and neck squamous cell carcinomas [10-13]. However, the role of RFCs in cancer initiation and progression remains unclear. In the current study, we investigated the expression levels of *RFC4* in CRCs, and determined the potential biological function of RFC4 in CRC.

## Materials and methods

### Data mining

The expression of RFC4 mRNA in CRC tissues was obtained by The Cancer Genome Atlas (TCGA), Gene Expression Omnibus databases (GEO) and BioGPS database (Biogps.org). For TCGA analysis, we queried The Cancer Genome Atlas [http://tcga-data.nci.nih.gov/] for colon cancer patients. “Level 3 of Exp-Gene” files from COAD Data Matrix Datasets were downloaded and used to extract mRNA expression for *RFC4*. For GEO data analysis, we downloaded and analyzed data publicly available from the GEO [http://www.ncbi.nlm.nih.gov/geo/; 82 samples from GSE9348 and 105 samples from GSE5206 and 64 samples from GSE8671]. “Series Matrix” Files were used to extract mRNA expression for *RFC4*. The genes amplification folds were analyzed and visualized by Graph Prism6 (GraphPad software Inc., San Diego, CA).

### Patients and tissue specimens

First, to detect the expression dynamics of *RFC4* in CRC, 30 paired freshly frozen specimens and 49 paired formalin-fixed, paraffin-embedded (FFPE) specimens from primary CRC tissues and patient-matched normal colonic tissues were obtained from the 6^th^ Affiliated Hospital of Sun Yat-sen University (Guangzhou, China). Each normal colonic tissue was acquired from a distance of at least 10 cm from the tumor margin. Second, to determine the clinical relevance of RFC4 in CRC, 331 FFPE CRC tumor specimens resected between January 2000 and December 2006 were obtained from the pathology archives at the 1^st^ Affiliated Hospital of Sun Yat-sen University (Guangzhou, China) for tissue microarray analyses. Among the 331 patients, 91 patients (27.5%) had been censored as death and 97 patients (29.3%) had developed distant metastasis or local recurrence after a median follow-up of 73.0 months (range 1–122). None of the patients had received neoadjuvant chemotherapy or radiotherapy, and the status of all samples was confirmed by pathologists after resection. Tumor tissues were staged according to the 7^th^ edition of the Union for International Cancer Control Tumor-Node-Metastasis (TNM) staging system. Written informed consents for using tissue samples for research purposes were obtained from all patients. This study was approved by the Institutional Review Board of the 1^st^ and 6^th^ Affiliated Hospital of Sun Yat-Sen University and all clinical and pathological data of the enrolled patients were collected from the Institutional Review Board approved CRC database, which was maintained by specialists.

### Cell lines

The human CRC cell lines, SW480 and DLD1, were obtained from the Culture Collection of the Chinese Academy of Science (Shanghai, China) and cultured in RPMI 1640 media. All media were supplemented with 10% fetal bovine serum, 100 U/ml penicillin, and 100 μg/ml streptomycin. The cells were cultured at 37°C in a humidified atmosphere of 95% air and 5% CO_2_.

### RNA extraction and quantitative PCR

All RNA extractions were performed using the Trizol Reagent (Life Technologies, Carlsbad, CA, USA) according to the manufacturer’s protocols. For first-strand complementary DNA synthesis, total RNA was reverse-transcribed with an oligo-dT primer using the RevertAid™ First Strand cDNA Synthesis Kit (Fermentas, Burlington, Canada). Quantitative PCR (qPCR) reactions were performed with an ABI PRISM® 7500 Fast Real-time PCR System (Applied Biosystems, Foster City, CA, USA) and a SYBR Green qPCR Mix Kit (Takara, Japan). β-actin expression was used as the normalization control. The following temperature profiles were used: initial heating at 95°C for 10 min, followed by 40 cycles of denaturation at 95°C for 15 s, annealing at 60°C for 60 s, and extension at 95°C for 15 s. The primers used were:

*RFC4* forward: 5′-GCGGAAACCTGAGGAACGAGCC-3;

*RFC4* reverse: 5′-TGGCAGCTACTCCTCGATCCTTG-3;

*β-actin* forward: 5′-TGGATCAGCAAGCAGGAGTA-3;

*β-actin* reverse: 5′-TCGGCCACATTGTGAACTTT-3.

Data were analyzed using the 2^-ΔΔCt^ method.

### Western blotting

Total cellular proteins were extracted from tissues or cells, separated by SDS-PAGE, and transferred onto a polyvinylidene difluoride membrane (Pall, New York, USA). Membranes were blocked with 5% nonfat milk in 1% Tween-PBS (PBST) and then probed overnight with anti-RFC4 rabbit polyclonal antibody (1:1000, Epitomics, Burlingame, CA, USA) or anti-ACTB antibody (1:1000, Proteintech, Chicago, IL, USA). After three washing steps of 10 min in PBST, membranes were incubated with species-appropriate fluorescently-conjugated secondary antibodies (1:10000 in PBST, LI-COR Biosciences, Lincoln, NE, USA) for 1 h at room temperature. The immunoreactive signals were detected using the two-color fluorescent western blotting Odyssey infrared imaging system (LI-COR Biosciences).

### Construction of tissue microarrays (TMAs)

To validate the overexpression of RFC4 and to define the clinical roles of RFC4 in CRC, two TMAs were constructed. The first contained tissue cores of 49 paired specimens of primary CRC tissues and matched normal colonic tissues. The second contained additional spots of 331 resected CRC specimens. For each case, two cores (1 mm diameter) were taken from the selected tumor area, and two additional cores (1 mm diameter) were taken from histologically confirmed normal adjacent colorectal mucosa to construct the TMAs using Tissue Array (Alphelys, MINIPORE, Plaisir, France). The TMAs were performed as we described previously [14].

### Immunohistochemistry (IHC) staining

The TMAs were deparaffinized in xylene and rehydrated with graded ethanol. Sections were then heated in antigen retrieval solution (EDTA, pH 9.0) for 20 min and incubated with 3% H_2_O_2_ for 10 min. The sections were then incubated with anti-RFC4 rabbit polyclonal antibody (1:800, Epitomics) at 4°C overnight. The sections were then treated with the secondary antibody for 15 min at room temperature and stained with 3, 3′-diaminobenzidine until brown granules appeared in the membrane, cytoplasm, or nucleus (Dako, EnvisionSystem/DAB-chromogen, Glostrup, Denmark). The sections were counterstained with hematoxylin for 2 min at room temperature. A negative control was employed by exchanging the specific primary antibody with non-immune serum immunoglobulins at a 1:200 dilution.

### Evaluation of IHC staining and selection of the optimal cutoff score

The immunoreaction was semi-quantitatively scored by assessing the extent and intensity of nuclear staining. Weak cytoplasmic staining was identified in some cases. The intensity of nuclear staining was scored as follows: negative (0), weak (1), moderate (2), and strong (3). The extent of nuclear staining was graded into quartiles according to the percentage of stained cells in each field: 0-25% (1), 26-50% (2), 51-75% (3) and 76-100% (4). The merged overall score (staining intensity plus staining extent) was then calculated. IHC staining was scored independently by two blinded pathologists to the clinical data (Huang and Fan).

To generate a logical cutoff score for further analysis, the nuclear RFC4 expression scores of 49 paired CRC and matched normal colonic tissues were subjected to the receiver operating characteristic curve (ROC) analysis. The score localized closest to the point at both maximum sensitivity and specificity (0.0, 1.0) was selected as the cutoff score leading to the greatest number of specimens which were correctly classified as tumor or normal.

### RFC4 siRNA gene knockdown

To knockdown endogenous RFC4 expression, cell lines were transfected with 20 nM of duplexed siRNA (RiboBio, Guangzhou, China) using Lipofectamine® RNAiMAX Reagent (Life Technologies). Duplex siRNAs included the following: RFC4 siRNA #1 (sense 5′-CAACUCAGCUCGUCAAUCAdTdT-3, antisense 3′-dTdTGUUGAGUCGAGCAGUUAGU-5); RFC4 siRNA #2 (sense 5′-GACCAAGGAUCGAGGAGUAdTdT-3, antisense 3′-dTdTCUGGUUCCUAGCUCCUCAU-5); RFC4 siRNA #3 (sense 5′-GAAAGUGAUUACAGACAUUdTdT-3, antisense 3′-dTdTCUUUCACUAAUGUCUGUAA-5). Duplexes of siRNA with non-specific sequences were used as scrambled siRNA. SW480 and DLD1 cells were transfected with RFC4 siRNA or scrambled siRNA according to the manufacturer’s instructions.

### Cell proliferation assays

Cells were plated at equal densities in 96-well plates for 24 h and then transiently transfected with the indicated siRNAs (6 biological replicates per condition). Cell proliferation was assessed using the Vybrant MTT Cell Proliferation Assay Kit (Invitrogen) according to the manufacturer’s instructions. Absorbance was measured at 490 nm.

The effect of RFC4 on proliferation was also tested by the 5-ethynyl-2′-deoxyuridine (EdU) cell proliferation assay using the Cell-Light™ EdU DNA Cell Proliferation Kit (RiboBio, Guangzhou, China). Briefly, cells (1 × 10^4^) were seeded in each well of 96-well plates for transfection with RFC4 siRNA or scrambled siRNA. After incubation at 37°C under 5% CO_2_ for 96 h, 50 μM EdU was added and the cells were incubated for another 2 h. Cells were then fixed with 4% paraformaldehyde and stained with Apollo® Dye Solution for proliferating cells. Nucleic acids in all cells were stained with Hoechst 33342. The extent of cell proliferation was calculated according to the manufacturer’s instructions. Images were taken using a fluorescence microscope (Olympus FSX100). All experiments were performed in triplicate.

### Cell cycle analysis

Cells were trypsinized, washed with cold PBS, and fixed overnight in 70% ethanol pre-chilled to −20°C. For the measurement of DNA content, cells were stained with a propidium iodide solution (50 mg/mL propidium iodide, 100 mg/mL RNase A, 0.05% Triton X-100 in PBS) and incubated at 37°C in the dark for 30 min. DNA content was assessed using a BD FACSCanto II flow cytometer (BD Biosciences) with ModFit LT software (Verity Software House, Topsham, ME).

### Statistical analyses

Overall survival (OS) time was measured from the date of surgery to the date of death due to any cause, or the date of the last clinical follow-up. Disease-free survival (DFS) time was defined as the time from surgery to the date confirmed local failure, distant metastases, or death due to disease or treatment, whichever occurred first. Kaplan-Meier survival curves were evaluated by the log-rank test. Univariate Cox regression analyses were performed to determine the prognostic significance of individual clinicopathologic variables. Multivariate analysis to test statistical independence and the significance of multiple predictors was performed by stepwise Cox analysis with backward selection using the Akaike Information Criterion. Wilcoxon matched-pairs, signed-rank test were used to assess IHC score within groups (cancer and matched normal tissues). Chi-square tests were used to compare dichotomized RFC4 groups and baseline clinicopathologic factors. Mann–Whitney *U* test were used to compare continuous variables and categorical variables. The level of significance for all tests was defined as *P* <0.05. All statistics were performed using SPSS v. 17.0 (SPSS, Inc., Chicago, IL).

## Results

### RFC4 is overexpressed in CRC

**Figure 1:**
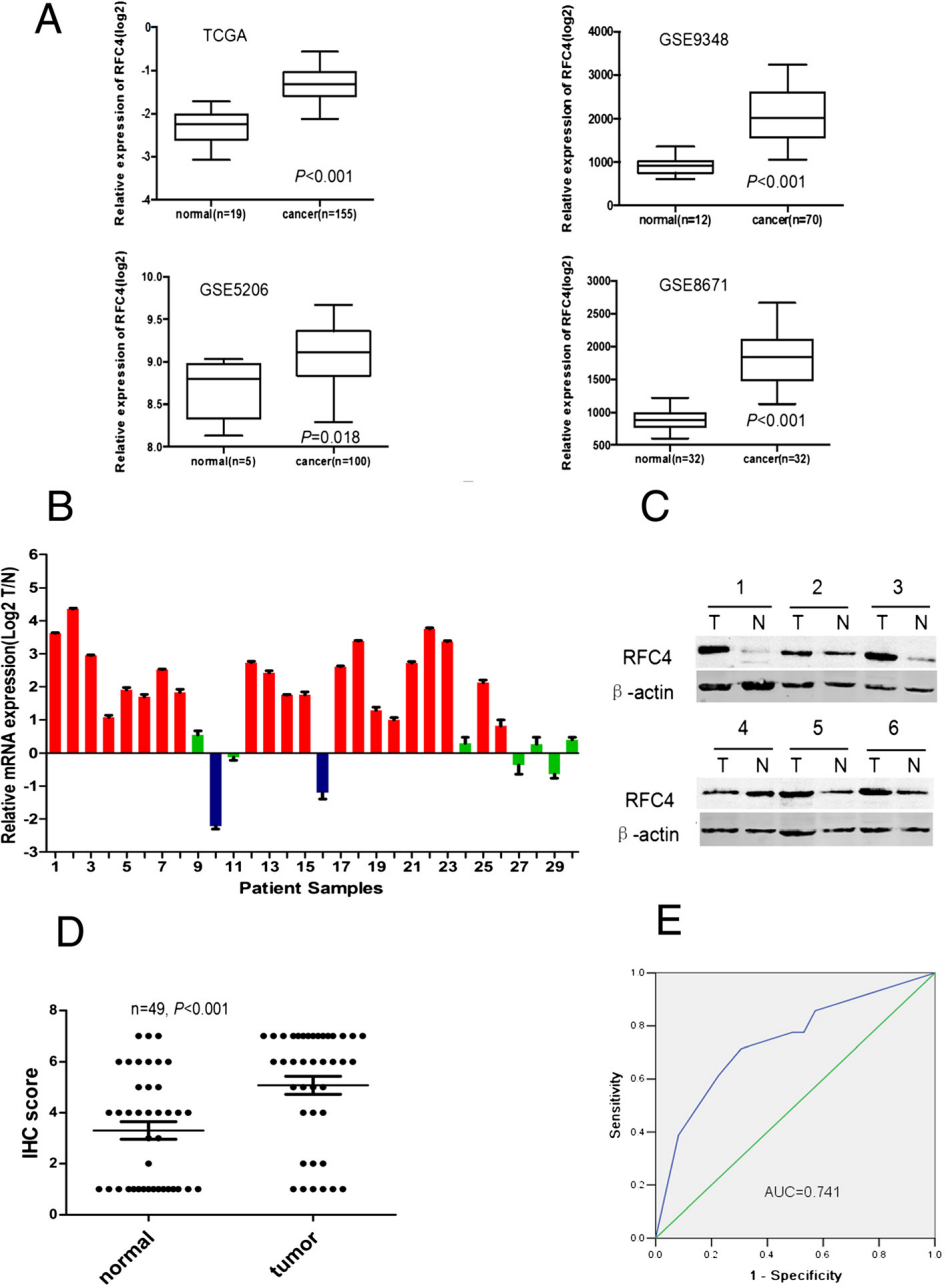
***RFC4*** **is overexpressed in CRC tissues. (A)** RFC4 mRNA levels were assessed from The Cancer Genome Atlas (TCGA) and the Gene Expression Omnibus databases (GEO, GSE9348, GSE5206 and GSE8671). Bounds of the boxes denote interquartile range; solid lines denote median, whiskers denote 95% and 5% confidence limits, respectively. **(B)** Expression of RFC4 mRNA in 30 paired CRC tumor and adjacent non-tumor tissues. Each bar is the log_2_ value of the ratio of *RFC4* expression levels between CRC (T) and matched normal tissues (N) from the same patient. Red bars (bar value ≥1) indicate high RFC4 mRNA levels. Blue and green bars represent low and normal expression of *RFC4* mRNA, respectively. Each sample was analyzed twice. **(C)** Western blots for RFC4 in matched-pairs of CRC tumor and non-tumor tissues. High RFC4 protein expression in CRC is seen in 12 of 16 patients. **(D)** Distribution of RFC4 immunoreactivity scores in tumor and paired-non-tumor tissues from the tissue microarray. CRC tissues exhibite higher RFC4 expression than normal colonic tissues. **(E)** ROC curves analysis to determine the cutoff score for the high expression of RFC4. The area under curve (AUC) is 0.741, and the cutoff point of RFC4 for overexpression is 4.5.

First, we analyzed the data from the TCGA and GEO for mRNA expression in CRC patients. *RFC4* expression was higher in colon cancer than normal colon tissue (Figure 1A). Furthermore, by using the BioGPS Gene Expression Atlas, we found that *RFC4* expression was highly expressed in colon cancer (Additional file 1: Figure S1).

To validate our finding, RNA was extracted from 30 paired CRC and adjacent normal tissues, and qPCR was done to measure the levels of RFC4 mRNA. RFC4 mRNA was significantly elevated in 21 of 30 CRC tissues, compared with patient-matched normal tissues (Figure 1B). Western blot analysis also revealed overexpression of RFC4 in 12 of 16 cancer tissues that had analyzed by qPCR, compared with normal colonic tissues (Figure 1C). Immunohistochemistry on TMA contained an independent set of 49 paired CRC and matched-normal tissues showed CRC tend to exhibit a higher level of RFC4 expression than normal colonic tissue (*P* <0.05) (Figure 1D). The clinical features of these patients were summarized in Table 1. Together, these data confirm the up-regulation of *RFC4* in CRC.

**Table 1:** **Clinical characteristics of the patients included in analyses of RFC4 expression evaluated by qPCR, Western and IHC**

**Characteristics**	**qPCR cases (n = 30 pairs)**	**Western cases (n = 16 pairs)**	**IHC cases (n = 49 pairs)**
**Age (years)** Mean ± SD	57.8 ± 10.9	56.8 ± 11.2	64.0 ± 12.3
**Sex**			
Female	6 (20.0%)	5 (31.2%)	16 (32.7%)
Male	24 (80.0%)	11 (68.8%)	33 (67.3%)
**Tumor location**			
Colon	16 (53.3%)	9 (56.3%)	23 (46.9%)
Rectal	14 (46.7%)	7 (43.7%)	26 (53.1%)
**Differentiation**			
Well	5 (16.7%)	2 (12.5%)	6 (12.2%)
Moderate	21 (70.0%)	12 (75.0%)	37 (75.6%)
Poorly	4 (13.3%)	2 (12.5%)	6 (12.2%)
**TNM stage**			
I	3 (10.0%)	2 (12.5%)	6 (12.2%)
II	19 (63.3%)	9 (56.3%)	18 (36.8%)
III	8 (26.7%)	5 (31.2%)	22 (44.9%)
IV	0 (0%)	0 (0%)	3 (6.1%)

For further survival analysis, ROC curve analysis of 49 paired specimens was performed to determine a reasonable cutoff point for RFC4 overexpression (Figure 1E). A score of 4.5, which maximizes the sum of sensitivity and specificity according to the ROC curves, was determined as the cutoff point.

### RFC4 expression correlates with a poor prognosis in CRC

**Figure 2:**
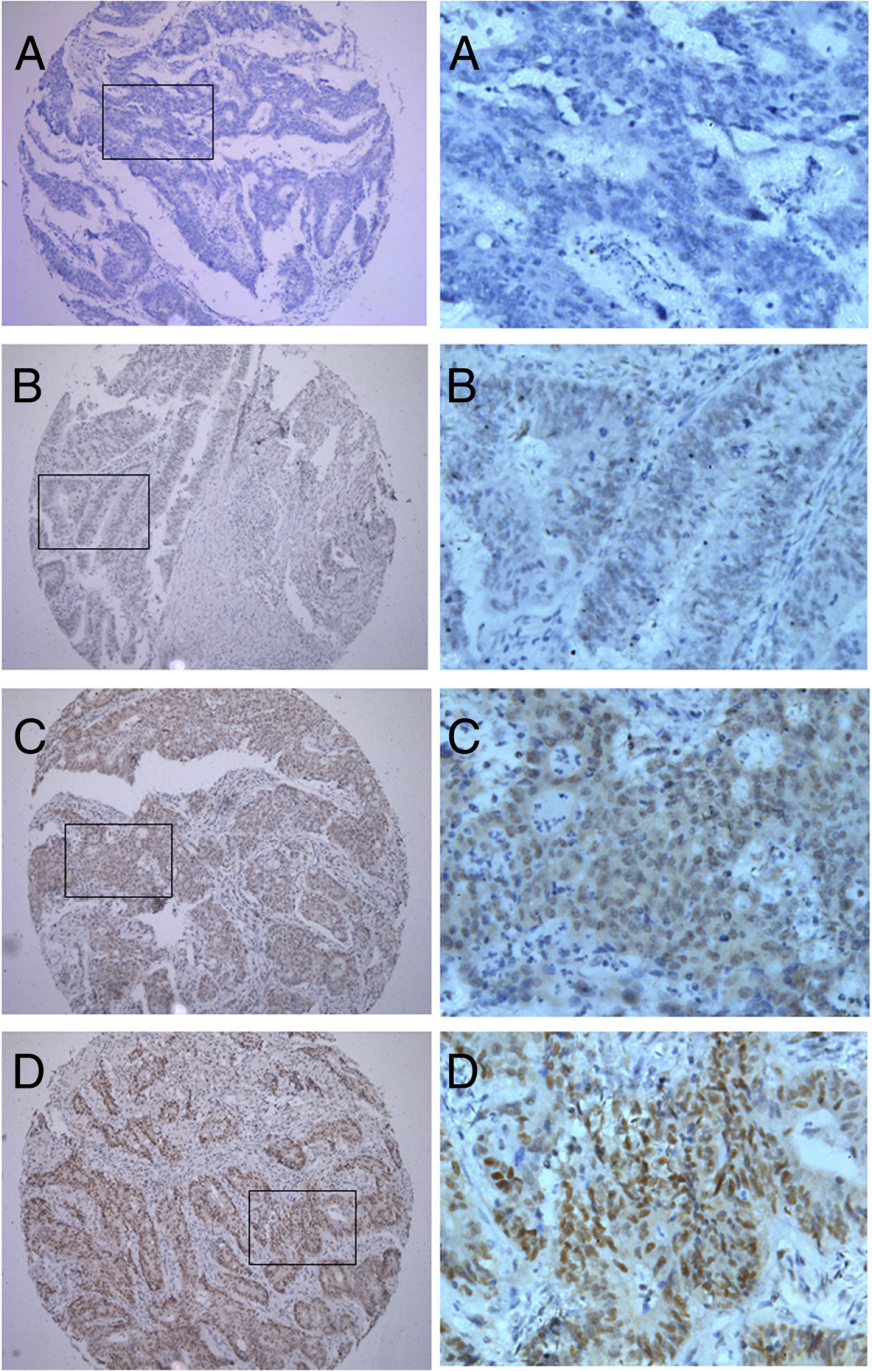
**Expression pattern of RFC4 in CRC by immunohistochemistry.** The low power photomicrographs (left side) in each section show the full tissue spot (×100), while the high power photomicrographs (right side) show details of RFC4 expression (×400). Nuclear staining is considered RFC4 expression positive. **(A)** negative nuclear staining; **(B)** weak nuclear staining; **(C)** moderate nuclear staining; **(D)** strong nuclear staining.

**Figure 3:**
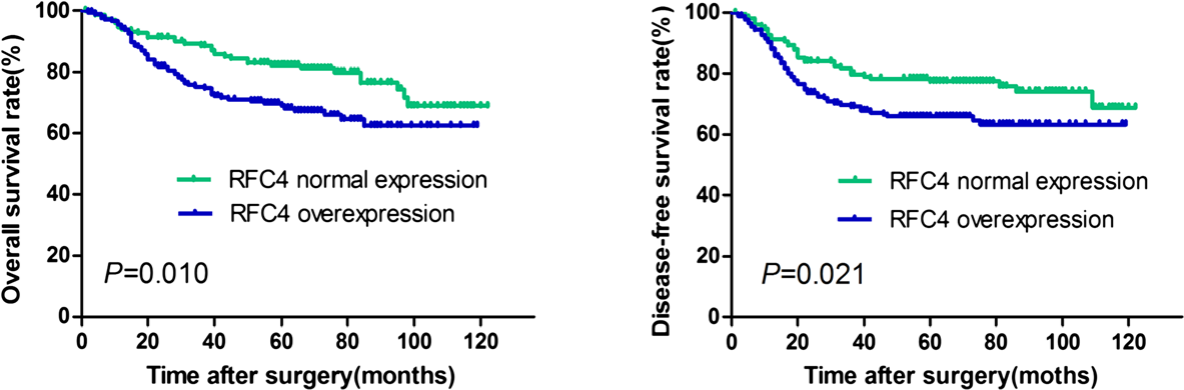
**The prognostic value of RFC4 in CRC.** Kaplan-Meier analysis of overall survival and disease- free survival was based on RFC4 expression in all 331 patients. High expressing patients (n = 179) are significantly less likely to survive compared with normal expressing patients (n = 152). *P* values were determined by the log-rank test.

The potential significance of RFC4 protein levels in CRC was examined by IHC on the TMA consisting of tissues from 331 CRC tumors, which were divided into a normal expression group and an overexpression group based on the above cutoff value. Dichotomized groups were evaluated in relation to clinicopathologic factors and overall survival. The RFC4 IHC staining results are summarized in Table 2 and Figure 2. RFC4 expression positively correlated with differentiation and TNM stage (*P* <0.05; Table 2). Patients with poorly differentiated or advanced TNM stage CRC were frequently identified with RFC4 overexpression. No significant associations were noted between RFC4 expression and other clinicopathologic variables. In relation to clinical outcomes, high levels of RFC4 expression were significantly associated with poor OS and DFS by Kaplan-Meier analysis (Figure 3). The 5-year OS and 5-year DFS were 81% and 78%, respectively for normal RFC4 expression patients, while the high RFC4 expressing patients had 5-year OS and 5-year DFS of 67% and 65%, respectively. In addition, univariate analysis revealed that the extent of differentiation, CEA level, CA19-9 level, pN stage, pM stage, and RFC4 expression were risk factors for death in CRC. Further Multivariate analysis was performed using the Cox proportional hazards model for all of the significant variables in the univariate analysis, including differentiation, preoperative CEA level, preoperative CA19-9 level, TNM stage and RFC4 expression Table 3. The results showed that RFC4 expression was not an independent predictor of worse overall survival, which might attribute to that there were more stage III/IV patients (60.9%) having high expression levels of RFC4.

**Table 2:** **Association of RFC4 protein expression with clinicopathologic characteristics in CRC**

**Characteristics**	**Cases**	**RFC4 expression**		***P*** **value**
		**Normal expression**	**Overexpression**	
**Sex**				0.059
Female	147	59 (40.1%)	88 (59.9%)	
Male	184	93 (45.9%)	91 (54.1%)	
**Age**				0.301
≤60	167	72 (43.1%)	95 (56.9%)	
>60	164	80 (48.8%)	84 (51.2%)	
**Tumor location**				0.344
Colon	154	75 (48.7%)	79 (51.3%)	
Rectal	177	77 (43.5%)	100 (56.5%)	
**Differentiation**				0.004
Well/Moderate	299	145 (48.5%)	154 (51.5%)	
Poorly	32	7 (21.9%)	25 (78.1%)	
**Preoperative CEA level**				0.656
< 5 ng/ml	194	89 (45.9%)	105 (54.1%)	
≥ 5 ng/ml	111	48 (43.2%)	63 (56.8%)	
**Preoperative CA19-9 level**				0.560
< 37 U/ml	238	109 (45.8%)	129 (54.2%)	
≥37 U/ml	67	28 (41.8%)	39 (58.2%)	
**pT (invasion depth)**				0.479
T1	6	1 (16.7%)	5 (83.3%)	
T2	54	27 (50.0%)	27 (50.0%)	
T3	233	106 (45.5%)	127 (54.5%)	
T4	38	18 (47.4%)	20 (52.6%)	
**pN (lymph node metastasis)**				0.055
N0	197	99 (49.2%)	98 (50.8%)	
N1-2	134	53 (46.3%)	81 (53.7%)	
**pM (distant metastasis)**				0.795
M0	299	138 (46.2%)	161 (53.8%)	
M1	32	14 (43.8%)	18 (56.2%)	
**TNM stage**				0.036
I + II	193	98 (50.8%)	95 (49.2%)	
III + IV	138	54 (39.1%)	84 (60.9%)

**Table 3:** **Univariate and multivariate analysis for overall survival in CRC tissue microarray**

**Variable**	**Univariate analysis**		**Multivariate analysis**	
	**Hazard ratio (95% CI)**	***P***	**Hazard ratio (95% CI)**	***P***
**Sex**				
Female	1	0.950		
Male	1.013 (0.670-1.533)			
**Age**				
≤60	1	0.222		
>60	1.294 (0.855-1.958)			
**Tumor location**				
Colon	1	0.061		
Rectal	1.502 (0.982-2.298)			
**Differentiation**				
Well + Moderate	1	<0.001	1	0.016
Poorly	2.736 (1.593-4.699)		1.945 (1.132-3.340)	
**Preoperative CEA level**				
< 5 ng/ml	1	<0.001	1	0.022
≥ 5 ng/ml	2.480 (1.590-3.866)		1.741 (1.084-2.797)	
**Preoperative CA19-9 level**				
< 37 U/ml	1	<0.001	1	0.014
≥37 U/ml	2.553 (1.614-4.038)		1.825 (1.127-2.954)	
**pT**				
T1 + T2	1	0.169		
T3 + T4	1.532 (0.834-2.813)			
**pN**				
N0	1	<0.001		
N1-2	2.438 (1.609-3.696)			
**pM**				
M0	1	<0.001		
M1	7.502 (4.701-11.971)			
**TNM stage**				
I + II	1	<0.001	1	0.006
III + IV	2.460 (1.618-3.741)		1.933 (1.210-3.089)	
**RFC4 expression**				
Normal	1	0.011	1	0.366
High	1.745 (1.136-2.680)		1.246 (0.774-2.005)	

### Down-regulation of RFC4 inhibits DNA synthesis and proliferation of CRC cells

**Figure 4:**
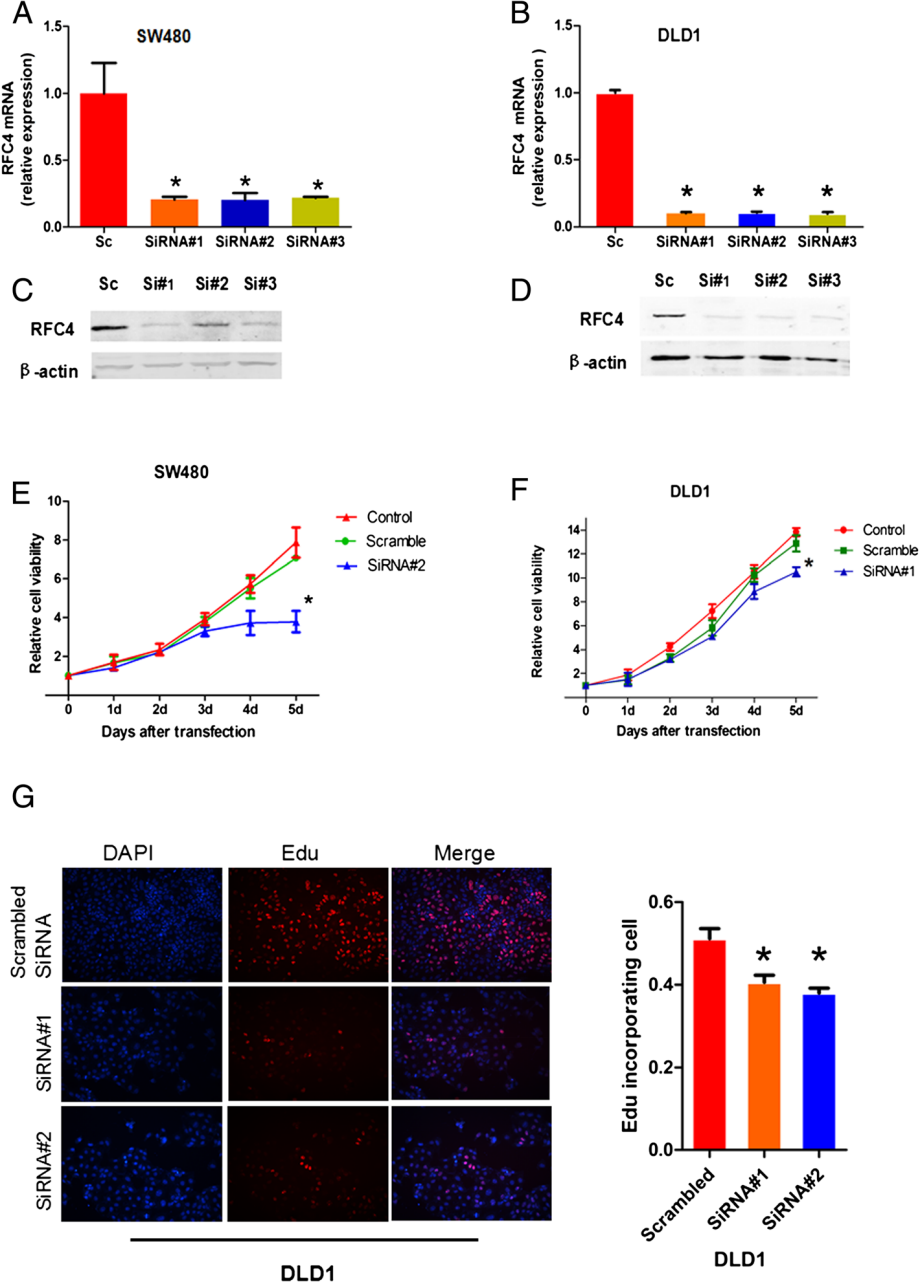
**Proliferation of CRC cells after transfection with RFC4-siRNA assessed by the MTT and EdU assays. (A)** RFC4 expression in SW480 cells following transfection with scrambled siRNA (Sc), RFC4-siRNA#1, −siRNA#2 or -siRNA#3. **(B)** RFC4 expression in DLD1 cells following transfection with scrambled siRNA (Sc), RFC4-siRNA#1, −siRNA#2 or -siRNA#3. **(C)** and **(D)** Western blots paralleling the treatments in panels A and B, respectively. **(E)** Cell growth in control and SW480 cells transfected with scrambled or RFC4-siRNA#2 as assessed by the MTT assay. **(F)** Cell growth in control and DLD1 cells transfected with scrambled or RFC4-siRNA#1 as assessed by the MTT assay. Transfection with RFC4 siRNA decreases cell proliferation in SW480 and DLD1 cells. **(G)** EdU incorporation assays for DNA synthesis in DLD1 cells transfected with scrambled siRNA or RFC4-siRNA. There is a significant reduction in the proliferation of DLD1 cells transfected with RFC-siRNA. *P <0.05.

Each of three distinct RFC4 siRNAs sharply reduced the expression of RFC4 mRNA and protein by 70% to 85% in both SW480 and DLD1 cells (Figure 4A, B, C and D). The number of viable SW480 and DLD1 cells 120 h after RFC4-siRNA transfection was significantly reduced (as assessed by the MTT assay) compared to that of the scrambled siRNA or non-siRNA groups (*P* <0.05) (Figure 4E and F). DNA synthesis in RFC4 knockdown tumor cells was significantly inhibited as compared to that in control tumor cells (*P* <0.05) (Figure 4G).

### Down-regulation of RFC4 induces S-phase arrest in CRC cells

**Figure 5:**
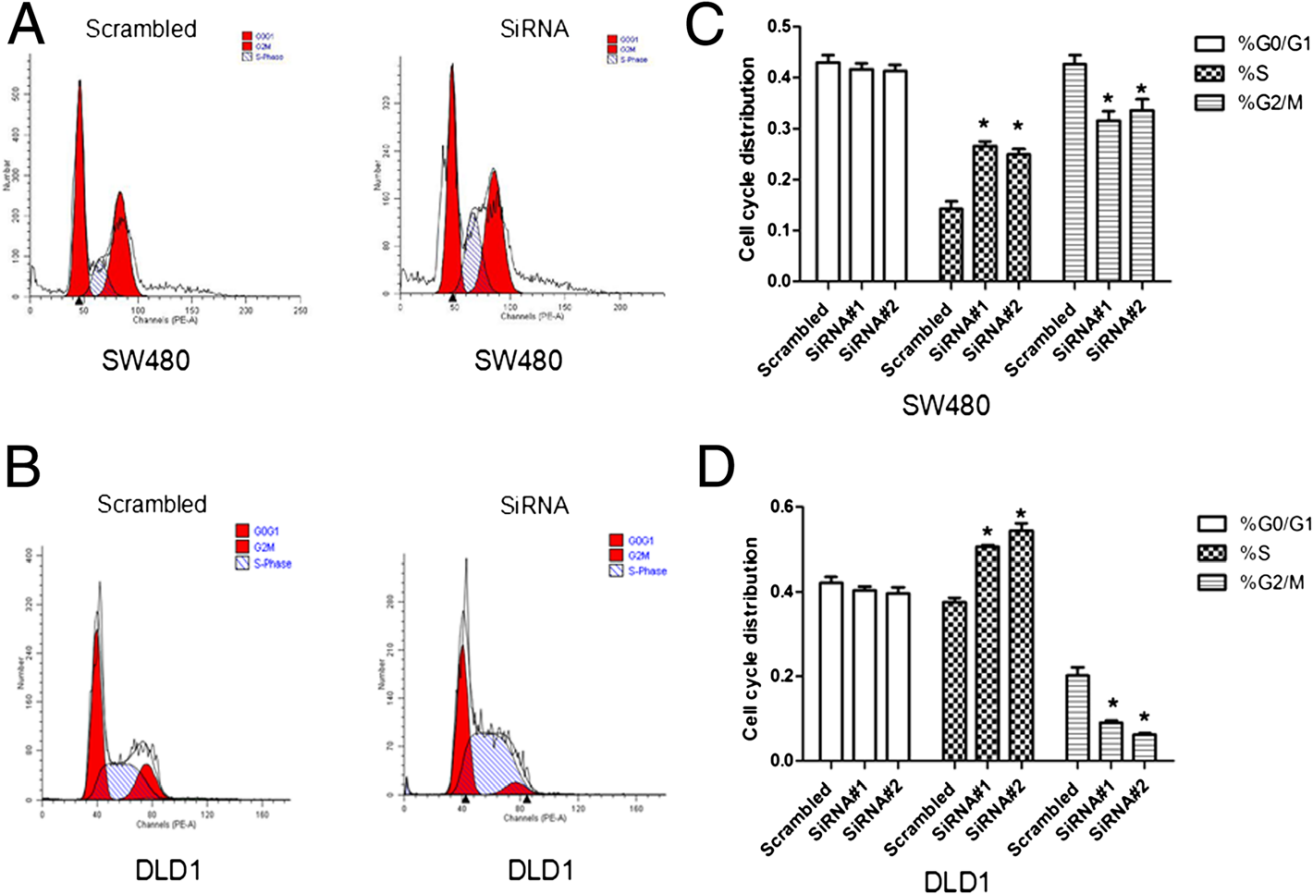
**Effect of RFC4-siRNA transfection on the cell cycle of SW480 and DLD1 cells.** Cell cycle distribution was analyzed by flow cytometry 48 h after transfection of scrambled siRNA or RFC4-siRNA in SW480 **(A)** and DLD1 **(B)** cells. The respective proportion of cells in the G0/G1 phase, S-phase, and G2/M phase following transfection with scrambled siRNA or RFC4-siRNA in SW480 **(C)** and DLD1 **(D)** cells. *P <0.05

To determine whether the inhibition of tumor cell growth was related to cell cycle regulation, the effects of RFC4 knockdown on cell cycle progression was investigated. Notably, the percentages of both SW480 and DLD1 cells in the S phase of the cell cycle were substantially increased following RFC4 knockdown (Figure 5). These data indicate that depletion of RFC4 induces S-phase cell cycle arrest, which might contribute to the suppression of cell proliferation.

## Discussion

RFC family members play important roles in eukaryotic DNA replication and the repair of DNA damage. Members of the RFC family may also be involved in several other biological processes such as S-phase checkpoint regulation, sister chromatid cohesion and genome maintenance [8,15,16]. Because cancer cells are actively replicating, RFCs are presumably deregulated in cancer. Indeed, this was evidenced by previous studies demonstrating that high expression of RFC family members occurs in diverse types of cancer. For example, amplification of *RFC3* is frequently found in esophageal adenocarcinoma [17], while RFC2 is overexpressed in nasopharyngeal carcinoma [18].

The current study showed that RFC4 is overexpressed in CRC and that increased RFC4 expression is significantly associated with poorly differentiated and advanced tumor TNM stage. Furthermore, high RFC4 expression predicts an unfavorable outcome in CRC patients. These findings demonstrate that RFC4 may play an oncogenic role in CRC. The vast majority of published studies on the RFC family have focused on their biological functions. However, the role of RFCs in the development and progression of cancer remains unclear. Thus, we further investigated the relationship between RFC4 and the proliferation of cancer cells. The knockdown of RFC4 expression in CRC cell lines by siRNA resulted in a significant decrease in cell proliferation assessed by both the MTT and Edu assays. This result further supported our contention that RFC4 is involved in DNA replication in cancer cells.

Our data showed that knockdown of RFC4 by siRNA significantly increases the number of CRC cells in the S-phase. This finding is consistent with the effect of RFC4 in hepatocellular carcinoma and breast cancer [10,19] and indicates that RFC4 acts as a sensor in cell cycle checkpoint control. Knockdown of RFC4 contributes to the decrease in intra-S phase checkpoints and allowing more cells with DNA damage to enter the S-phase where they are unable to combine with EdU and subsequently fails to prevent cells with DNA damage from entering mitosis. In addition, RFC4, together with PCNA, is required for large loop DNA repair synthesis [20]. Other studies demonstrated that RFC recruits DNA polymerase delta to sites of nucleotide excision repair but is not required for the recruitment of PCNA [21]. Regardless, the DNA damage checkpoint is critical for repair in response to DNA damage caused by some chemotherapeutic agents. Interestingly, a previous study revealed that the downregulation of RFC4 can enhance the cytotoxic effects of doxorubicin and camptothecin in hepatocellular carcinoma cells [19]. Together, these findings indicate that decreasing RFC4 may sensitize colon cancer cells to DNA damaging agents that are commonly used in chemotherapy.

## Conclusion

In summary, our results reveal that the overexpression of *RFC4* commonly occurs in CRC, and that a high level of RFC4 is associated with poorly differentiated and late TNM stages in patients with CRC. Higher levels of RFC4 protein expression correlate with a worsened overall survival in CRC. Yet, we appreciate that these findings need to be validated in independent sets of clinical samples in future study. The underlying mechanism appears to involve RFC4, which promotes cancer cell proliferation, while reducing the RFC4 levels induces the formation of an enriched population of CRC cells in the S phase and a decrease in CRC cells proliferation.

## Additional file

Additional file 1
**Screenshot of the profile of the human RFC4 gene within the BioGPS online portal.** All data used for this study are available through the BioGPS database (Biogps.org). Gene expression profile is displayed as a bar chart.

## Abbreviations

CRC: Colorectal cancer
RFC4: Replication factor C4
KRAS: Kirsten rat sarcoma viral oncogene
EGFR: Epidermal growth factor receptor
PCNA: Proliferating cell nuclear antigen
TNM: Tumor-Node-Metastasis
TMA: Tissue microarray
IHC: Immunohistochemistry
PBST: Phosphate buffer solution-Tween
EDTA: Ethylenediaminetetraacetic acid
EdU: 5-ethynyl-2′-deoxyuridine
OS: Overall survival
DFS: Disease-free survival
