# Supplementary figures and images for: Levels of human replication factor C4, a clamp loader, correlate with tumor progression and predict the prognosis for colorectal cancer
Source: J Transl Med. 2014 Nov 19;12:320. doi: 10.1186/s12967-014-0320-0 (PMC4256821; doi:10.1186/s12967-014-0320-0)

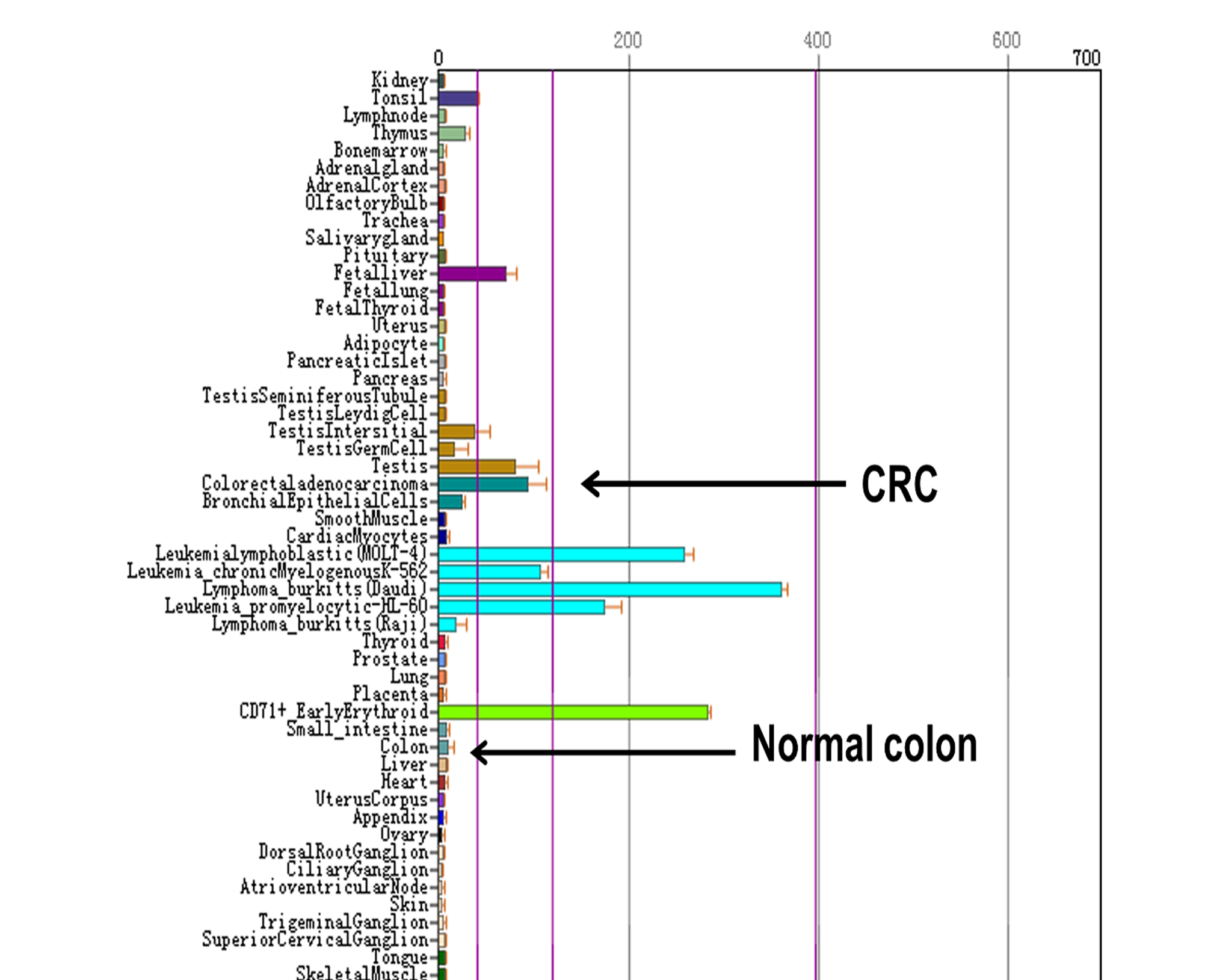

Supplement: Additional file 1 — Screenshot of the profile of the human RFC4 gene within the BioGPS online portal. All data used for this study are available through the BioGPS database (Biogps.org). Gene expression profile is displayed as a bar chart. [file 12967_2014_320_MOESM1_ESM.tiff]
